# Supplementary material for: Multiple phosphorylation of the Cdc48/p97 cofactor protein Shp1/p47 occurs upon cell stress in budding yeast
Source: Life Sci Alliance. 2023 Jan 24;6(4):e202201642. doi: 10.26508/lsa.202201642 (PMC9874129; doi:10.26508/lsa.202201642)
Supplement: Supplementary file 1 [file LSA-2022-01642_Supplemental_Data_1.docx]

Supplementary Materials & Methods

Phos-tag gel electrophoresis gel preparation and running

Equipment:

- **Mini-PROTEAN Tetra Cell gel electrophoresis system** (Bio Rad #1658003EDU)
- **Trans-Blot® Turbo™ Transfer System** (Bio Rad 1704150)
- **Transfer packs: Midi format, 0.2µm nitrocellulose** (BioRad # 1704159).

Reagents and buffers:

- **1 M MnCl2** aliquots in distilled water, stored at -20°C in the dark. Keep frozen when not being used. Made up using tetrahydrate stock (MnCl2·4H2O MW=197.91, used 1.5g in 7.5ml)
- **5mM Phos-tag acrylamide** stored at -20°C in the dark.
- **Running buffer for Phos-tag**
- **Transfer buffer for Phos-tag**
- **1.5 M Tris pH 8.6**
- **1 M Tris pH 6.8**
- **10% SDS solution**
- **Isopropanol**
- **10% APS (Ammonium Persulfate) solution** stored at 4°C
- **N,N,N′,N′-Tetramethyl ethylenediamine (TEMED)**
- **40% 29:1 Acrylamide:Bis-Acrylamide Solution** (ND, EC-852)

**Running buffer for Phos-tag:**

25 mM Tris, 192 mM Glycine, 0.1% SDS.

Always use fresh for inner chamber, outer chamber buffer can be re-used.

For 1 L of 5x concentrated:

- 15.15 g Tris base
- 72 g Glycine
- 50 ml of 10% SDS solution

**Transfer buffer for Phos-tag:**

48 mM Tris, 39 mM Glycine, 20% methanol.

For 1 L of 5x concentrated:

- 29.07 g Tris base
- 14.6 g Glycine.

To make 1L of 1x transfer, mix 200 ml 5x with 200 ml methanol + 800 ml miliQ H2O.

Gel preparation:

- Gels can be prepared day before use, stored dark at 4°C. Use 1mm plates.
- Prepare mixtures at room temp in falcon tubes.
- Add all components except TEMED, only add the TEMED immediately prior to pouring the resolving gel (and stacking gel later on).
- Pour resolving gel first up to the height of the green band (exactly 5 ml). Add a layer of isopropanol to flatten the meniscus.
- Allow the resolving gel to polymerise for at least 80 minutes. Then remove isopropanol by pouring onto tissue and wicking.
- Add TEMED to the stacking gel mix, then fill stacking gel to top, remove bubbles with a micropipette and insert the 15 well gel comb. Allow ~45 mins to polymerise at room temp.

6% Resolving gel recipe:

| **Component** | **Vol for 2 gels** |
| --- | --- |
| ddH2O | 7.8 ml |
| 1.5 M Tris pH 8.6 | 3.5 ml |
| Acrylamide (40%) | 2.2 ml |
| 10% SDS | 140 µl |
| 5 mM Phos-tag | 140 µl |
| 1 M MnCl2 | 1.4 µl |
| 10% APS | 70 µl |
| TEMED | 14 µl |

Stacking gel recipe:

| **Component** | **Vol for 2 gels** |
| --- | --- |
| ddH2O | 3.8 ml |
| 1M Tris pH 6.8 | 620 µl |
| Acrylamide (40%) | 500 µl |
| 10% SDS | 50 µl |
| 10% APS | 42.5 µl |
| TEMED (when ready) | 5 µl |

Gel loading

- Fill central portion of gel tank with fresh running buffer, can use recycled running buffer for outside.
- Whenever using protein ladder (2-5µl per lane), spike this into a duplicated sample (preferably non-expressing lysate) loaded at equal volume and concentration to other samples, to avoid distortion between lanes.

Gel running, washing and transfer

- Run gel at room temperature, 115V constant (~95 mins total).
- Trim all gel edges and remove the stacking gel.
- Wash gel 1x 10 mins in Phos-tag transfer buffer containing 10 mM EDTA, finish with rinsing 2-3x briefly in Phos-tag transfer buffer (without EDTA). Leave the gels soaked in excess transfer buffer.
- Set up bottom section of transfer pack in the centre of the transfer cassette (grab from the tabs labelled ‘bottom’), use roller to flatten and remove bubbles from the membrane.
- Making sure there are no bubbles or foam on the gels, place the gels foot-to-foot (lower MW facing each other) towards the centre of the pack on top of the membrane. Try to minimise gaps between the gels. Lay the gel gently from one side to the other to avoid bubbles forming. Don’t use the roller directly on the gel.
- Place the top section of the transfer pack and use the roller to remove bubbles. In particular, make sure the area with the gels is perfectly flat.
- Transfer for **12 mins** at 2.5 A constant (25 V limit)
- Remove the transfer pack from the cassette before trimming the membrane with a scalpel.

LC-MS/MS Proteomics sample preparation

**IP lysis buffer recipe:**

100 mM NaCl, 50 mM Tris 7.5, 1% Triton

- 1 ml 5M NaCl
- 2.5ml 1M Tris 7.5
- 0.5 ml Triton
- Top up with miliQ to 50ml total volume.

Then take the base buffer and add the following supplements fresh on the day of experiment,

For 20 ml, add:

- + PIC tablet (add fresh, 2 tablets mini)
- + phosstop tablet (add fresh, 2 tablets)
- + 20 mM NEM (add fresh - 400 µl of 1M stock in ethanol stored at -20°C)
- + 1 mM PMSF (add fresh - 200 µl of 0.1M sigma stock in ethanol)

Day 1: Culturing, lysis and pulldown

1. [Set up 10ml starter cultures of each yeast strain the day before the experiment.]
2. In the morning, make 2 x 30 ml YPD culture in 50 ml falcon tubes for each replicate (6 = 12 total). Use 800 uL of starter culture to give approx 0.2 OD.
3. Grow yeast in flasks at 30°C for 4 hours to ~0.4 OD
4. Treat directly with 30ul diluted rapamycin (200 nM final), don’t adjust cultures. Incubate for 2h.
5. During treatment, prepare 20 ml lysis buffer with all supplements, cool and keep on ice (store in fridge overnight).
6. Centrifuge cultures 3750 rpm 5 mins at room temp.
7. Pool 2 pellets for each replicate by adding 800 ul of cold water and resuspending, transferring to fresh 1.5 ml tube. Spin at 8000 rpm for 1 min, then wash pellet again with 1ml cold water.
8. Resuspend each pellet in 500 uL lysis buffer and add to pre-cooled 2ml tube containing 500 ul glass beads.
9. Bash for 3 x 30 s, with 5 min rest in between. After final bash, can put tubes on ice.
10. Spin 2 mins max speed (13,000 rpm) at 4°C, transfer supernatant into a new 1.5ml tube using gel loading tips.
11. Spin 10 mins max speed at 4°C, keep supernatant after transferring to a fresh tube. Expect to recover just over approx. 350 ul volume for each sample.
12. Quantify protein using nanodrop (A280) and dilute to equal concentrations (expected concentration approx. 5ug/ul):
    1. In a new tube, prepare 500 uL sample at 2.5 ug/ul.
    2. Take a 30 ul aliquot of input sample into a new tube and freeze at -80°C.
    3. Keep the remaining 470 uL on ice for IP (1.175 mg protein).
13. Take 120 ul bead slurry of anti-FLAG M2 magnetic beads (20 uL slurry per pulldown) into tube using cut P200 tip. Apply to magnet and remove supernatant using vacuum aspirator.
14. Wash beads 2x in 600 ul buffer, vortexing each time before applying to magnet and then inverting the rack several times to wash all beads towards the magnet.
15. Finally resuspend the beads by adding 950 ul buffer, vortexing and transferring 150 ul of bead suspension to each IP using a fresh P1000 tip. Vortex thoroughly before each transfer. (Note, resuspension in 50ul excess to prevent running out of beads, also bead volume provides further excess).
16. Ensure lids are properly sealed, place samples on rotator in the coldroom and cover with foil. Leave overnight.

Day 2: IP elution and sample trapping, overnight digestion

1. Apply samples to magnet and rotate tubes, then take 30 uL flow-through sample for each and store at -80°C. Remove the remainder of flow-through using vacuum aspirator.
2. Wash each set of beads 4x with 300 uL supplemented lysis buffer each time (500 uL for last wash). As before, apply to magnet and rotate tube to wash all beads towards magnet.
3. Prepare elution solution of 150 ng/ul 3xFLAG peptide in supplemented lysis buffer (13.5 uL of 10mg/ml stock with 886.5 uL buffer. Add 130 ul to each set of beads as elution volume. Elute on rotator at 4°C for 1-2 hours.
4. Place samples on magnet and rotate rack to collect all beads. Transfer 100 uL of elution to a new 2 ml tube containing 100 uL of 2x SDS lysis buffer for S-trap (20% SDS, 100 mM TEAB pH 8.5 – note should not need to change pH from 1M stock. Use MiliQ water for everything and LC/MS grade reagents).
5. Take 20 uL of IP sample to another new tube and store at -80°C for subsequent western blot analysis.
6. For samples mixed with the 2x SDS lysis buffer, continue the S-Trap mini protocol from step 5 onwards (Reduce, alkylate etc.) steps taken are listed here, see that document for more specific details. Remember that before loading on column, volumes need to be adjusted for sample volume of 200 uL (instead of usual 46 uL).
7. Reduce: add 2 uL of 500 mM TCEP to final conc of 5 mM (from glass vial, use undiluted). Vortex and then incubate samples at 55 deg C for 15 mins.
8. Prepare Iodoacetamide stock for sample alkylation. Weigh approx. 100 mg and dissolve in water for 500 mM stock, volume needed should be close to 1ml (MW = 185).
9. Add 8.7 uL of 500 mM Iodoacetamide to each sample, vortex and incubate at room temp for 10 mins, covered in foil/protect from light.
10. Prepare 12 % phosphoric acid (141 uL of 85% phosphoric acid + 859 uL water). Add 5 uL of to each sample and vortex.
11. Prepare 25 ml of binding/wash buffer (100 mM TEAB 90% methanol, use 2.5 ml of 1M TEAB and 22.5 ml of methanol. Note that protocol says pH 7.55 TEAB, but pH was not adjusted).
12. Add 1552 uL of binding buffer to each sample. Mix, load 400 uL at a time of each onto clean S-trap columns. (800 uL can be added each time as long as the collection tube is emptied after each spin).
13. Spin columns at 4000 x g for 30 s each time. Repeat until all sample has been run through its respective column.
14. Add 400 uL binding/wash buffer and centrifuge 4000 x g for 30 s. Repeat the wash 3 more times, emptying collection tube when needed.
15. Empty the collection tube and spin the column 4000 x g for 1 minute to dry/remove residual wash buffer.
16. Transfer the S-trap column to a clean 2 mL sample tube for digestion and subsequent elution.
17. Dissolve 100 ug trypsin in 100 uL of 50 mM TEAB for a 1ug/ul stock.
18. Use 10 ug trypsin per column in a total volume of 125 ul. Add to column, making sure no air bubbles, and cap the tubes and place them on a rack with wet tissue near the edge, wrapping with cling film to maintain humidity. The aim is to avoid the columns drying out. Digest overnight in an incubator at 37°C.

Day 3: peptide elution and drying.

1. Remove columns from incubator, don’t centrifuge them yet.
2. Add 80 uL of elution buffer 1 to each column (50 mM TEAB in water, remainder stored in fridge bottom shelf). Centrifuge 4000 x g 1 minute to collect (don’t discard the elution! All eluates will be combined.)
3. Add 80 uL of elution buffer 2 (0.2 % formic acid in water, made by mixing one 1ml ampule with 500 ml water. Remainder stored in fridge). Centrifuge again to collect eluate.
4. Add 80 uL of elution buffer 3 (50% acetonitrile, made by mixing 1:1 with miliQ water, remainder in fridge). Centrifuge again to collect all eluate.
5. Take 5% of total eluate into new tube (18.25 uL) to dry separately. Dry all samples on speedvac, 01.0 vac pressure, 45°C, 1 hour or for as long as needed. Store dried peptides at -80°C or proceed to LC-MS/MS.

LC-MS/MS

The 5% of total eluate samples were analysed by LC-MS/MS as follows:

- Dried samples were resuspended in 12.5 µL of 5% formic acid in water
- 10 uL were injected on an Ultimate 3000 coupled to a Thermo Exploris 480

Peptides were analysed by nanoflow-LC-MS/MS using an Exploris 480 Mass Spectrometer (Thermo Scientific) coupled to a Dionex Ultimate 3000. Samples were injected on a 100 μm ID × 20 mm trap (Acclaim PepMap 100 C18 LC, #164564) and separated on a 75 μm × 50 cm nano LC column (EASY-Spray LC Columns #ES803). All solvents used were HPLC or LC-MS Grade (Millipore). Peptides were loaded for 5 minutes at 5 μL/min using 0.1% TFA, 2% Acetonitrile in Water. The column was conditioned using 97% Buffer A (0.1% FA, in Water) and 3% Buffer B (0.1% FA, 20% Water in Acetonitrile). The separation was performed on a multistep gradient at 300 nL/min from 5.4 to 7% buffer B in 2 minutes, then to 25% in 43 minutes, 35% in 5 minutes and 95% in 3 minutes. The column was then washed with 95% buffer B for 2 minutes and equilibrated for 4 minutes with 3% buffer B.

Full MS scans were acquired from 350 Th to 1200 Th at resolution 60000 at 200 Th, with an AGC target of 300% and an automatic maximum injection time. MS/MS scans were acquired in HCD mode with a normalized collision energy of 30% and resolution 15000 using a Top 2 s method, with an AGC target of 100%, a maximum injection time of 100 ms and an isolation window of 1.2 Th. The MS/MS triggering threshold was set at 1E4 and the dynamic exclusion of previously acquired precursor (+/- 10 ppm) was enabled for 30 s.

Peptides were searched against Uniprot Swissprot Saccharomyces cerevisiae (strain ATCC 204508 / S288c) Database containing isoforms (released 5 July 2021) and quantified using MaxQuant 1.6.17.0.

All parameters were left to default, with the exception of:

- Modifications:
  - Variable: Oxidation (M), Acetyl (Protein N-term), Deamidation (NQ)
  - Fixed: Carbamidomethyl (C)
- Label-free quantification was enabled (with default parameters)
- Match between runs was enabled (with default parameters)

Data analysis was performed in Python 3.9

- Reverse, only identified by site, potential contaminants were removed
- Proteins identified with strictly less than 2 Razor + unique peptides were removed
- Proteins with less than 2 valid values in at least 1 condition were removed
- LFQ intensities were imputed using a left censored approach with q downshift of 1.8 and width of 0.3
- Significance was assessed using 2 Sample Welch T-test with a P-value threshold of 0.05 and fold change threshold of 2 and -2
- P-values were corrected using Benjamini-Hochberg correction.

Table of primers used

| **Primer name** | **Sequence** |
| --- | --- |
| Shp1 5FL His fw | CATTGAAGGACGCTGATCTGCTGAACTCCGTTGTCGTGCAAAGATGGGCACGTACGCTGCAGGTCGAC |
| Shp1 5FL His rev | ATATTAAGTTGAAGTCTTTTCCCGTTTCTGTTTTTGTATATTTATGCTCAATCGATGAATTCGAGCTCgt |
| Npl4-5f fw | TCTCATCGGCTGGTTGGCAAACGTTAGTCATGATCCTTCAGGAAAGCGGCCGTACGCTGCAGGTCGAC |
| Npl4-5f rv | CGCATTTCTATTTTTTTAATCTAAAACCTCAAATCTTACAGTCTCTCCTAATCGATGAATTCGAGCTCGT |
| Ufd1-5f fw | ATTCGTCCAAGTCCAAGGCTCCCAAAAGCCCGGAAGTAATCGAAATTGACCGTACGCTGCAGGTCGAC |
| Ufd1-5f rv | ATATATCTGTCAAAGACACTATATTGTTGTATACTAGTATATATCTTTTAATCGATGAATTCGAGCTCGT |
| Ubx2-5f fw | TGGAAGCACTGGATGAAGAAGATGAAGAAGATGAGGAAAATGAAGAACAACGTACGCTGCAGGTCGAC |
| Ubx2-5f rv | AGAAACTCTTTGTACGCGTTTGTCGTTTTTAACGATATGCTATTTTATCAATCGATGAATTCGAGCTCGT |
| Ubx3-5f fw | TTAGCGGCATATATCCATCAGGAAATATTGTAATGGAACGCCTGGACGAACGTACGCTGCAGGTCGAC |
| Ubx3-5f rv | TAAACTTTAGAATAAACGTTTTGAGATGACTATTTTTGAAATTCCTTTCAATCGATGAATTCGAGCTCGT |
| Ubx4-5f fw | TCAAAAAGACTTTAAATAGAGTACCTAAATGGATGAAATTAAGTAAAAAACGTACGCTGCAGGTCGAC |
| Ubx4-5f rv | TACATATTTGTAATAAAGTTAAAAGAGGAGACTGTTTAGCTTTGTATTTAATCGATGAATTCGAGCTCGT |
| Ubx5-5f fw | GCTTAAAAAATAGTTCTTTACTACTTGAGAAGCTTGACCCTGAAATAGAACGTACGCTGCAGGTCGAC |
| Ubx5-5f rv | GCTTACATACCTAATTACATCTAGGTACCTGCCACCATACAATTTGTTTAATCGATGAATTCGAGCTCGT |
| Ubx6-5f fw | CTAATACCAAAGTTCGTACATTAGGTTACTCTAATAATAACGGCAACAATCGTACGCTGCAGGTCGAC |
| Ubx6-5f rv | AAGAAAATATGTGTGAATAACCAAATAGGAAATAAACAAAAGCACATTTAATCGATGAATTCGAGCTCGT |
| Ubx7-5f fw | TTAATGGTAATAATGTCCATTTAGAAAAGAAAAAGGACGAAGATAAAAAACGTACGCTGCAGGTCGAC |
| Ubx7-5f rv | ATTCACGTCTAATACTAGTCATAAATGGAGTTGAAGCCATTAACTACTTAATCGATGAATTCGAGCTCGT |
| Cdc48 5FL His fw | CAGGTGCTGCATTTGGTTCTAATGCGGAGGAAGATGATGATTTGTATAGTCGTACGCTGCAGGTCGAC |
| Cdc48 5FL His rev | AATGACTTGAATTTACGATTTAAAATAAAAATATACCTGGCATATAACTAATCGATGAATTCGAGCTCgt |
| Shp1 S108A fw | TCTACCGCTCCTGAACCAACCAAGG |
| Shp1 S108A rv | TTCAGGAGCGGTAGAACCTTTCCTTTTCAAG |
| Shp1 S231+2A fw | GAAGCGGCACCTGCGGAGGTTCC |
| Shp1 S231+2A rv | CGCAGGTGCCGCTTCACCCGGGATAGGAGAT |
| Shp1 S315A fw | CTAGGAGCTCCTATCCCGGGTGAA |
| Shp1 S315A rv | GATAGGAGCTCCTAGTCTTTGGCCCTGG |
| Shp1 S321A fw | GGTGAAGCGTCACCTGCGGAG |
| Shp1 S321A rv | AGGTGACGCTTCACCCGGGATAGG |
| Shp1 S322A fw | GAATCGGCACCTGCGGAGG |
| Shp1 S322A rv | CGCAGGTGCCGATTCACCCGG |
| Shp1 T331A fw | AATGAGGCACCCGCTGCTCAG |
| Shp1 T331A rv | AGCGGGTGCCTCATTCTTTGGAACC |
| Met4 5FL His fw | AAAAAGCTGAAAGCTTAAAGAAGCAAATTTTTGAGAAGGTTCAGAAAGAACGTACGCTGCAGGTCGAC |
| Met4 5FL His rev | CACGTATATATATATATATATATAATTAAACTGTATAGTCTGTTATTTTAATCGATGAATTCGAGCTCgt |
| 3xHA in GPDEco Rv | gcttgatatcgaattTCActgagcagcgtaatctggaac |
| p416 Dld1 fw | cgggctgcaggaattATGTTGTGGAAGCGTACTTG |
| Dld1-3xHA int rev | aacatcgtatgggtaCCTGTAATCATTAGCGGGCTC |
| Dld1-3xHA int fw | GAGCCCGCTAATGATTACAGGtacccatacgatgttcctgac |
| p416 Y134C fw | cgggctgcaggaattATGACTGGATACCCTGACG |
| Y134C-3xHA int rev | aacatcgtatgggtaAACCTTTCCACTCAGGTAATCTT |
| Y134C-3xHA int fw | AAGATTACCTGAGTGGAAAGGTTtacccatacgatgttcctgac |
| Ubx4 p416 fw | cgggctgcaggaattATGCCTATGGTTACCGTGAAG |
| Ubx4-3xHA int Fw | AGAGTACCTAAATGGATGAAATTAAGTAAAAAAtacccatacgatgttcctgac |
| Ubx4-3xHA int Rev | aacatcgtatgggtaTTTTTTACTTAATTTCATCCATTTAGGTACTCT |
| Ubx4 398-9AA fw | ACGCTGCAGCTATCAAAAAGACTTTAAATAGAGTACC |
| Ubx4 398-9AA rv | TGATAGCTGCAGCGTTATCAGAAGGGC |
| Ubx4 S104A fw | AGAGATGCCGTGGTTAAAGAAATGCCATC |
| Ubx4 S104A rv | AACCACGGCATCTCTTCCTGGGATTTG |
| Ubx4 S264A fw | GAGCAAGCAGCCAACAACTTGCCAAAG |
| Ubx4 S264A rv | GTTGGCTGCTTGCTCTCTAAGTCTCTTTG |
| Ubx4 S344A fw | TTTGGCGCTAAAACAATGCTGCTTTTTGAAAC |
| Ubx4 S344A rv | TGTTTTAGCGCCAAATTCCAGGTCATCC |
| Ubx4 T398A fw | AACGCTGCAAGTATCAAAAAGACTTTAAATAGAGTACCTA |
| Ubx4 T398A rv | GATACTTGCAGCGTTATCAGAAGGGCC |
| Ubx4 S399A fw | GCTACAGCTATCAAAAAGACTTTAAATAGAGTACCTAAATGGATG |
| Ubx4 S399A rv | TTTGATAGCTGTAGCGTTATCAGAAGGGC |
| Shp1-p416GPD-Fw | cgggctgcaggaattATGGCGGAAATACCTGATGA |
| Shp1-p416GPD-Rv | gcttgatatcgaattTCATGCCCATCTTTGCACG |
| Shp1-3xHA int Rv | aacatcgtatgggtaTGCCCATCTTTGCACGA |
| Shp1-3xHA int Fw | TCGTGCAAAGATGGGCAtacccatacgatgttcctgac |
| Mpk1-6xHis-Fw | CATCATCACCATCACCACTAGctcgagtcatgtaattagttatgtc |
| Mpk1-6xHis-Rv | GTGGTGATGGTGATGATGAAAATATTTTCTATCTAATCCAAACTCCAGC |
| Shp1 full fw | acaaaagctggagctcagttGAAATAAAGGACTGGCCAACAAT |
| Shp1 full rv | ttgggtaccggccgcaaattTGGACAGGCTAAAATAACGTTGA |
| Shp1 - gRNA - F | GATCCTGGAAGAGGTTTTAGATTAGTTTTAGAGCTAG |
| Shp1 - gRNA - R | CTAGCTCTAAAACTAATCTAAAACCTCTTCCAG |
| Shp1 valid seq - F | CAAGAAGCCTTCTCCACCAAC |
| Shp1 valid seq - R | CAGTGTTCGCATTTGATGTCAC |
| Shp1 PAM G-C - F | GTTTTAGATTAGGcTCAACCATCGACGCAGCAGA |
| Shp1 PAM G-C - R | GGTTGAgCCTAATCTAAAACCTCTTCCAGTAAACC |
| Shp1-NatMX6 - F | ATATATAAGAAACGTCGGTAGCACAACAATTAACTCATTATTTAGGTATGtaaggcgcgccagatctgtt |
| Shp1-NatMX6 - R | ATATTAAGTTGAAGTCTTTTCCCGTTTCTGTTTTTGTATATTTATGCTCAtgcaggttaacctggcttatcga |
| GFP forward primer | atgtctaaaggtgaagaattattcact |
| GFP reverse primer | tttgtacaattcatccataccatgg |
| ATG8 gene + linker + GFP overlap Fwd | gatgaattgtacaaaGGtGGCAGTGGtGGCAAGTCTACATTTAAGTCTGAATATCCATTTG |
| ATG8 gene + 3'UTR + overlap with pRS413 Rv | tcccggcaattcgatAAATTCATGTTCGACATATAGATAGCA |
| ATG8 5' Fwd + overlap with pRS413 | cgataagcttgatGATATATCATCACCCGGAGGC |
| ATG 8 5' Rv + overlap with GFP | ttcacctttagacatGTCTCTAGTAATTATTTTATTATGATTTTCTCAACT |
| pRS413-ADH amplify around EcoRv site | atcgaattGCCGGGATC |
| pRS413-ADH amplify around EcoRv site | atcaagcttatcgataccgtc |
